# Results of an Early Access Treatment Protocol of Daratumumab Monotherapy in Spanish Patients With Relapsed or Refractory Multiple Myeloma

**DOI:** 10.1097/HS9.0000000000000380

**Published:** 2020-06-03

**Authors:** Adrián Alegre, Javier de la Rubia, Anna Sureda Balari, Cristina Encinas Rodríguez, Alexia Suárez, María Jesús Blanchard, Joan Bargay Lleonart, Paula Rodríguez-Otero, Andrés Insunza, Luis Palomera, María Jesús Peñarrubia, Rafael Ríos-Tamayo, Luis Felipe Casado Montero, Marta Sonia González, Anna Potamianou, Catherine Couturier, Huiling Pei, Henar Hevia, Iordanis Milionis, Maren Gaudig, María-Victoria Mateos

**Affiliations:** 1 Hospital Universitario de La Princesa, Madrid, Spain; 2 Hospital Dr. Peset and School of Medicine and Dentistry, Catholic University of Valencia, Valencia, Spain; 3 Institut Català d’Oncologia-Hospitalet, Barcelona, Spain; 4 Hospital General Universitario Gregorio Marañón, Instituto de Investigacion Sanitaria Gregorio Marañón, Madrid, Spain; 5 Hospital Universitario Gran Canaria Dr. Negrin, Las Palmas, Spain; 6 Ramón y Cajal University Hospital, Madrid, Spain; 7 Hospital Sont Llàtzer, Palma de Mallorca, Spain; 8 Clínica Universidad de Navarra, CIMA, IDISNA, CIBERONC, Pamplona, Spain; 9 Department of Hematology, Hospital Universitario Marqués de Valdecilla, Santander, Spain; 10 Hospital Clínico Universitario Lozano Blesa, Instituto de Investigación de Aragón, Zaragoza, Spain; 11 Hospital Clínico Universitario de Valladolid, Valladolid, Spain; 12 University Hospital Virgen de las Nieves, Granada, Spain; 13 Hospital Virgen de la Salud, Toledo, Spain; 14 Hospital Clínico de Santiago, Santiago, Spain; 15 Janssen-Cilag, Neuss, Germany; 16 Janssen-Cilag, Issy les Moulineaux, France; 17 Janssen Research & Development, Horsham, Pennsylvania, United States; 18 Janssen-Cilag Medical Affairs, Madrid, Spain; 19 EMEA Medical Affairs, Janssen-Cilag Pharmaceutical SACI, Athens, Greece; 20 University Hospital of Salamanca/IBSAL, Salamanca, Spain

## Abstract

Daratumumab is a human CD38-targeted monoclonal antibody approved as monotherapy for heavily pretreated relapsed and refractory multiple myeloma. We report findings for the Spanish cohort of an open-label treatment protocol that provided early access to daratumumab monotherapy and collected safety and patient-reported outcomes data for patients with relapsed or refractory multiple myeloma. At 15 centers across Spain, intravenous daratumumab (16 mg/kg) was administered to 73 patients who had ≥3 prior lines of therapy, including a proteasome inhibitor and an immunomodulatory drug, or who were double refractory to both. The median duration of daratumumab treatment was 3.3 (range: 0.03–13.17) months, with a median number of 12 (range: 1–25) infusions. Grade 3/4 treatment-emergent adverse events were reported in 74% of patients and included lymphopenia (28.8%), thrombocytopenia (27.4%), neutropenia (21.9%), leukopenia (19.2%), and anemia (15.1%). Common (>5%) serious treatment-emergent adverse events included respiratory tract infection (9.6%), general physical health deterioration (6.8%), and back pain (5.5%). Infusion-related reactions occurred in 45% of patients. The median change from baseline in all domains of the EQ-5D-5L and EORTC QLQ-C30 was mostly 0. A total of 18 (24.7%) patients achieved a partial response or better, with 10 (13.7%) patients achieving a very good partial response or better. Median progression-free survival was 3.98 months. The results of this early access treatment protocol are consistent with previously reported trials of daratumumab monotherapy and confirm its safety and antitumoral efficacy in Spanish patients with heavily treated relapsed or refractory multiple myeloma.

European Clinical Trials Database number: 2015-002993-19

## Introduction

Proteasome inhibitors (PIs) and immunomodulatory drugs (IMiDs) have improved clinical outcomes for patients with multiple myeloma (MM) over the past decade; however, the majority of MM patients will relapse or become resistant to available drug treatment and require subsequent therapy.^1–3^ Patients with relapsed and/or refractory MM (RRMM) have a particularly poor prognosis, with an increased risk of adverse events and death with additional treatment.^4^ Therefore, safe and effective therapies are needed to improve clinical outcomes for patients with RRMM.

Daratumumab is a human monoclonal antibody targeting CD38, a 45-kDa type II transmembrane glycoprotein that is highly expressed on MM cells.^5^ Daratumumab binds CD38 and induces tumor cell death through a direct on-tumor and immunomodulatory mechanism of action that consists of antibody-dependent cellular phagocytosis, complement-dependent cytotoxicity, antibody-dependent cell-mediated cytotoxicity, apoptosis, and clonal expansion of cytotoxic T cells.^6–10^

Daratumumab has demonstrated deep and durable responses as a monotherapy and superior clinical benefit across lines of therapy when combined with standard-of-care regimens for the treatment of MM.^11–19^ In a combined analysis of the phase 1/2 GEN501 study and phase 2 SIRIUS study after 36.6 months of follow-up, RRMM patients treated with daratumumab monotherapy achieved an overall response rate of 30.4%, with 13.5% of patients achieving a very good partial response (VGPR) or better and 4.7% of patients achieving a complete response (CR) or better.^20^ Deep responses were maintained over time in both studies, and the combined median overall survival was 20.5 months (95% confidence interval [CI], 16.6–28.1).^20^ Furthermore, daratumumab monotherapy demonstrated a favorable safety profile with no new safety signals identified with longer follow-up.^20,21^

Based on these findings, daratumumab was approved as a monotherapy in the United States and Europe for the treatment of RRMM.^22,23^ Daratumumab has since been shown to be effective and safe in combination with standard-of-care regimens vs standard-of-care alone for MM patients who have received ≥1 prior line of therapy and for transplant-ineligible newly diagnosed MM patients in ongoing phase 3 clinical trials, where daratumumab-based regimens have been reported to reduce disease progression or death by ≥44%, nearly double CR or better rates, and at least triple minimal residual disease–negativity rates.^13–18^ More recently, the addition of daratumumab to bortezomib, thalidomide, and dexamethasone during pre-transplant induction and post-transplant consolidation was shown to significantly improve stringent complete response (sCR) and minimal residual disease–negativity rates and to reduce the risk of disease progression or death by 53% in transplant-eligible newly diagnosed MM patients in Part 1 of the phase 3 CASSIOPEIA study.^19^

Despite the demonstrated benefit of daratumumab in patients with MM, not all patients are eligible for inclusion in these clinical trials or have access to commercially available daratumumab. The objective of this study was to provide early access to daratumumab for eligible RRMM patients who may reside in areas where daratumumab is not yet commercially available through local health care providers, who have not been enrolled in another daratumumab study, or who do not have access to another ongoing clinical study of daratumumab. Here, we present findings from the Spanish cohort of this multicenter, open-label, early access treatment protocol (EAP; MMY3010; ClinicalTrials.gov identifier: NCT02477891; EudraCT number: 2015-002993-19) of daratumumab monotherapy in patients with MM who received ≥3 prior lines of therapy, including a PI and an IMiD, or who were double refractory to a PI and an IMiD.

## Results

### Patient demographics and disposition

A total of 73 patients (91.3% of patients screened) were enrolled at 15 centers in Spain, all of whom received ≥1 dose of daratumumab. Patient demographics and baseline characteristics are shown in Table 1. The median age was 65 (range: 41–85) years, and 47.9% of patients were male. The majority of patients had a baseline Eastern Cooperative Oncology Group (ECOG) performance status score of 0 (39.7%) or 1 (43.8%).

**Table 1:**
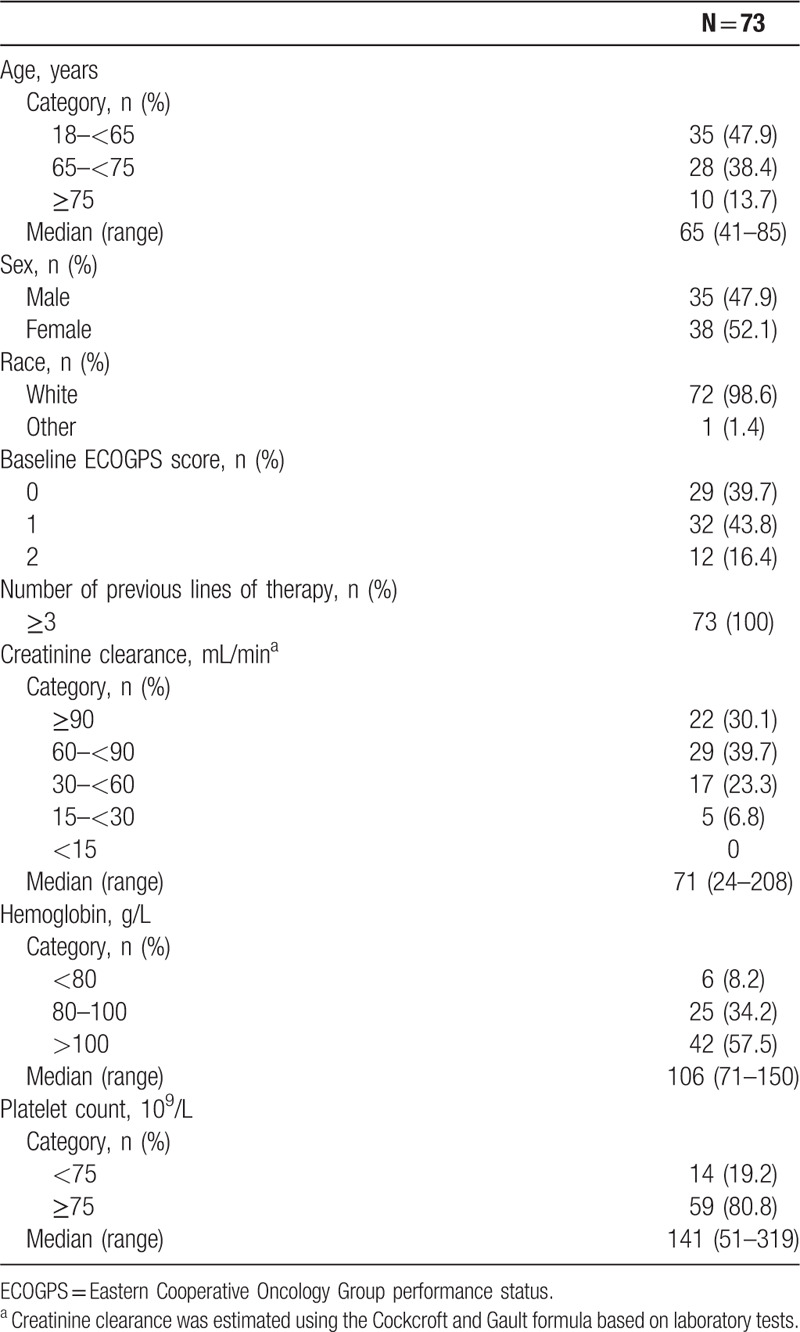
Patient Demographics and Baseline Characteristics.

At a median follow-up of 5.7 months, all patients had discontinued study treatment. Fourteen patients (19.2%) discontinued treatment due to market authorization/reimbursement and transitioned to commercially available daratumumab; these patients were no longer followed after transition. Other reasons for treatment discontinuations included progressive disease (61.6%), adverse event (12.3%), death (4.1%), lack of efficacy (lack of desired beneficial effect related to the therapy; 1.4%), and withdrawal by patient (1.4%).

### Treatment exposure

Patients received a median of 4 (range: 1–15) treatment cycles (Table 2), and 43.8% received ≥6 cycles of treatment. The median duration of daratumumab exposure was 3.3 months (range: 0.03–13.17 months), with a median number of 12 infusions (range: 1–25). Median durations of infusions were 7.1, 4.3, and 3.5 hours for the first, second, and all subsequent infusions, respectively. Common pre- and post-infusion medications included antihistamines (pre-infusion: 73 [100.0%] patients, post-infusion: 1 [1.4%] patient), corticosteroids (pre-infusion: 73 [100.0%] patients, post-infusion: 73 [100.0%] patients), and montelukast (pre-infusion: 11 [15.1%] patients, post-infusion: 1 [1.4%] patient).

**Table 2:**
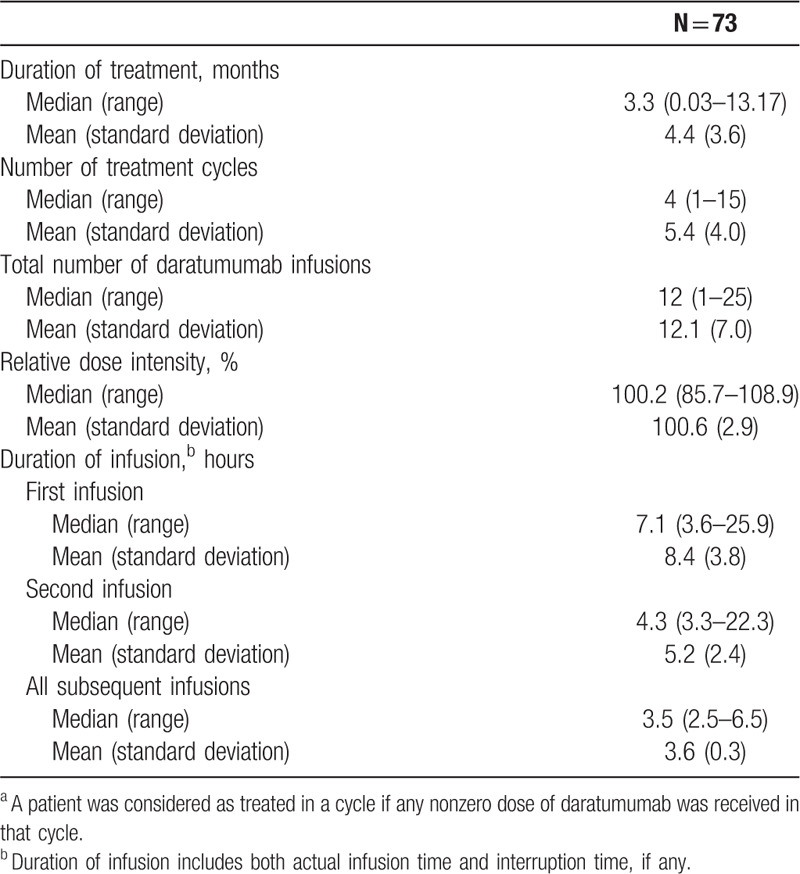
Treatment Exposure^a^ and Infusion Time.

### Safety

Grade 3/4 treatment-emergent adverse events (TEAEs) were reported in 54 (74.0%) patients (Table 3). The most frequently reported (>10%) grade 3/4 TEAEs were hematologic and included lymphopenia (28.8%), thrombocytopenia (27.4%), neutropenia (21.9%), leukopenia (19.2%), and anemia (15.1%). Fifteen (20.5%) patients discontinued therapy due to TEAEs; 3 (4.1%) were deemed daratumumab-related.

**Table 3:**
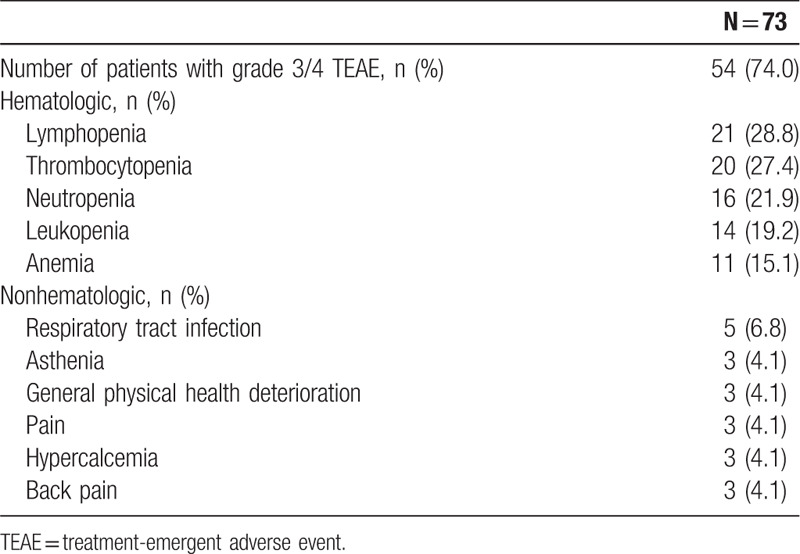
Most Common (>2 Patients) Grade 3/4 TEAEs.

Serious adverse events (SAEs) were reported in 35 (47.9%) patients, with grade 3/4 events occurring in 31 (42.5%) patients. Eleven (15.1%) patients had a fatal SAE (general physical health deterioration [n = 4], septic shock [n = 2], and multiple organ dysfunction syndrome, pelvic pain, pleural effusion, cardiac failure, respiratory tract infection, and hypercalcemia [each n = 1]); however, none of these events were daratumumab-related based on investigator assessment. The most common (>5%) SAEs were respiratory tract infection (9.6%), general physical health deterioration (6.8%), and back pain (5.5%). Respiratory tract infection was the most common grade 3/4 treatment-emergent SAE, occurring in 5 (6.8%) patients. Two (2.7%) patients had grade 3 SAEs that were at least possibly related to daratumumab therapy (Table 4). One patient withdrew from treatment due to grade 3 infusion-related reaction (IRR) SAEs (chest discomfort, dyspnea, and decreased oxygen saturation) on Cycle 1 Day 1 that resolved within a day of onset. The second patient recovered with sequelae from grade 3 back pain after 4 days but eventually withdrew from the study due to progressive disease.

**Table 4:**
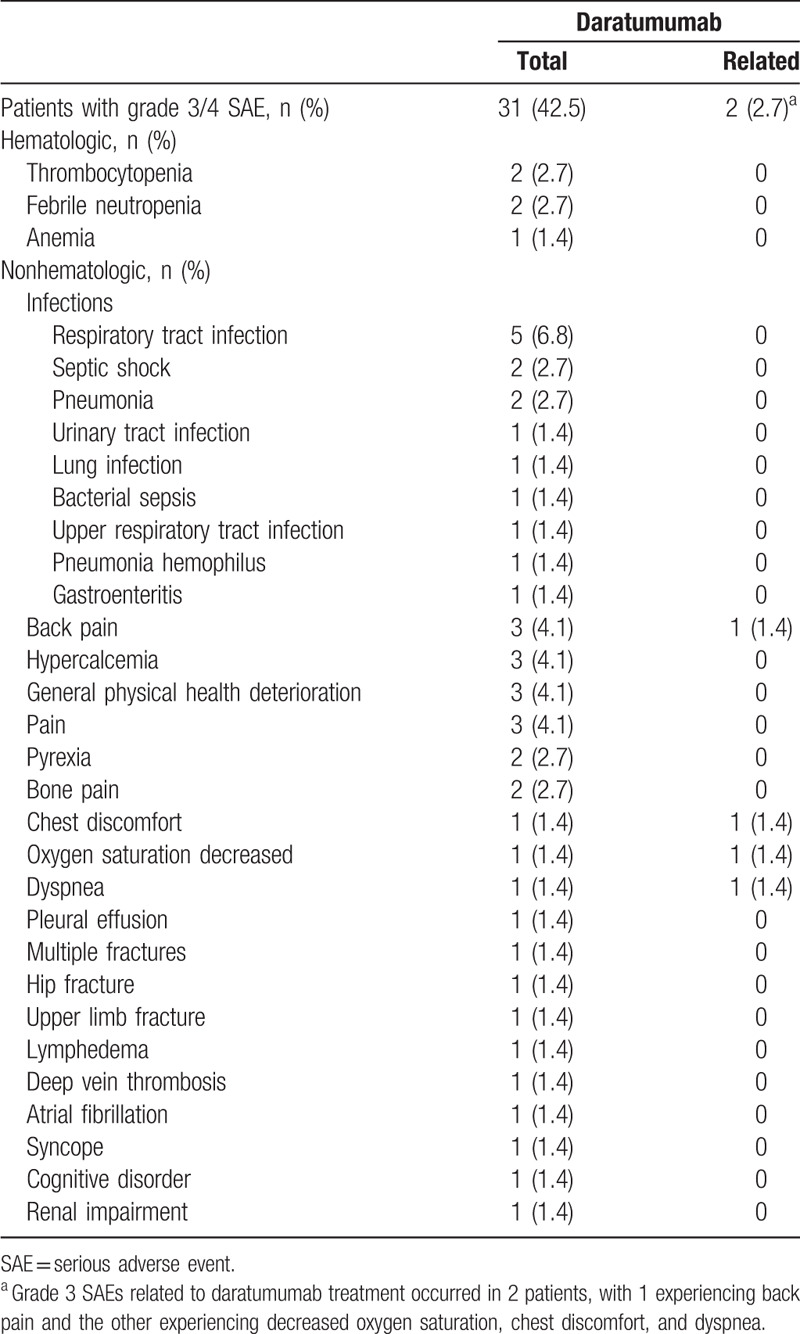
Grade 3/4 SAEs by Preferred Term and Relationship to Treatment.

IRRs were reported in 33 (45.2%) patients, were primarily grade 1 or 2, and occurred predominantly during the first infusion. One (1.4%) patient reported an IRR during the second infusion, and no IRRs were reported in subsequent infusions. The most common (>5%) IRRs were nasal congestion (12.3%), dyspnea (11.0%), nausea (11.0%), decreased oxygen saturation (6.8%), cough (5.5%), and throat irritation (5.5%; Table 5). Grade 3/4 IRRs occurred in 2 (2.7%) patients and included dyspnea, bronchospasm, chest discomfort, and decreased oxygen saturation (each 1.4%).

**Table 5:**
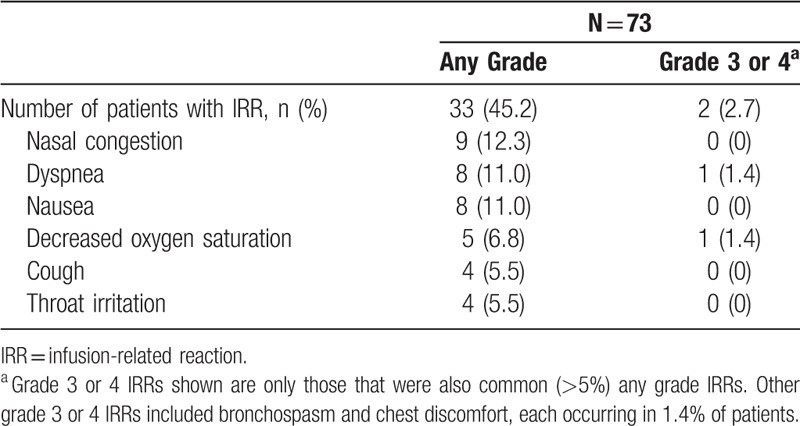
Most Common (>5%) IRRs.

### Efficacy and survival

**Figure 1:**
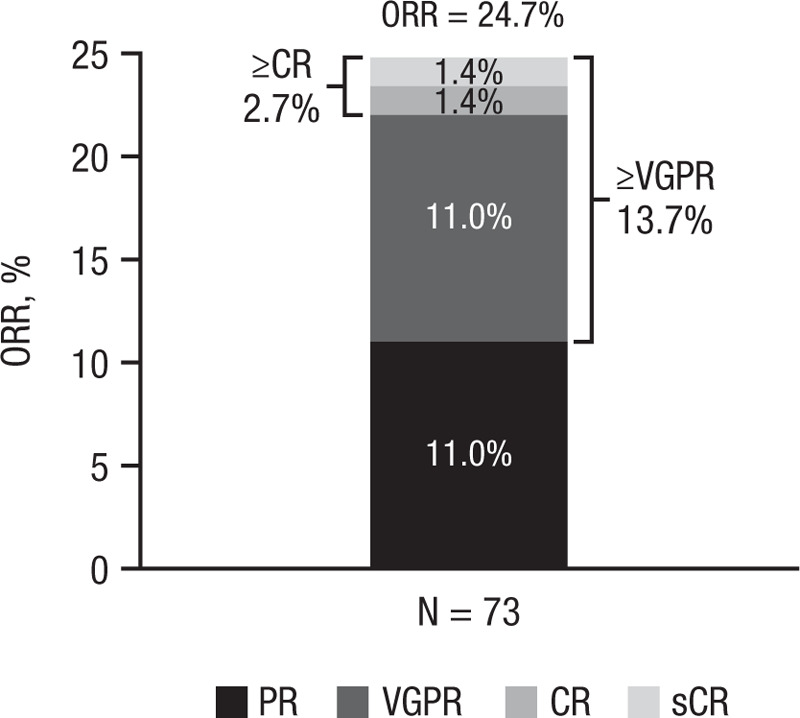
**Investigator-assessed objective disease response in Spanish RRMM patients**. RRMM = relapsed and/or refractory multiple myeloma, ORR = objective response rate, CR = complete response, VGPR = very good partial response, PR = partial response, sCR = stringent complete response. Note: individual response rates may not sum to total response rates due to rounding.

**Figure 2:**
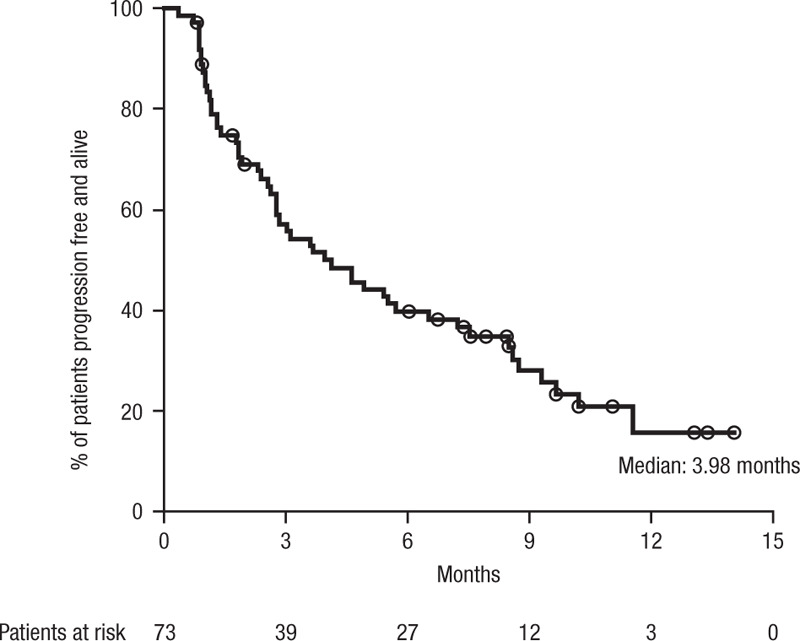
**Progression-free survival in Spanish patients with RRMM**. RRMM = relapsed and/or refractory multiple myeloma.

The investigator-assessed objective disease response (sCR + CR + VGPR + partial response [PR]) was 24.7% (Fig. 1). Best disease responses included 1 (1.4%) sCR, 1 (1.4%) CR, 8 (11.0%) VGPRs, and 8 (11.0%) PRs. Minimal response was achieved in 7 (9.6%) patients, and stable disease was observed in 17 (23.3%) patients. Median progression-free survival (PFS) was 3.98 (95% CI, 2.8–6.5) months (Fig. 2), and the 6-month PFS rate was 39.7% (95% CI, 28.2–50.9).

### Patient-reported outcomes

**Figure 3:**
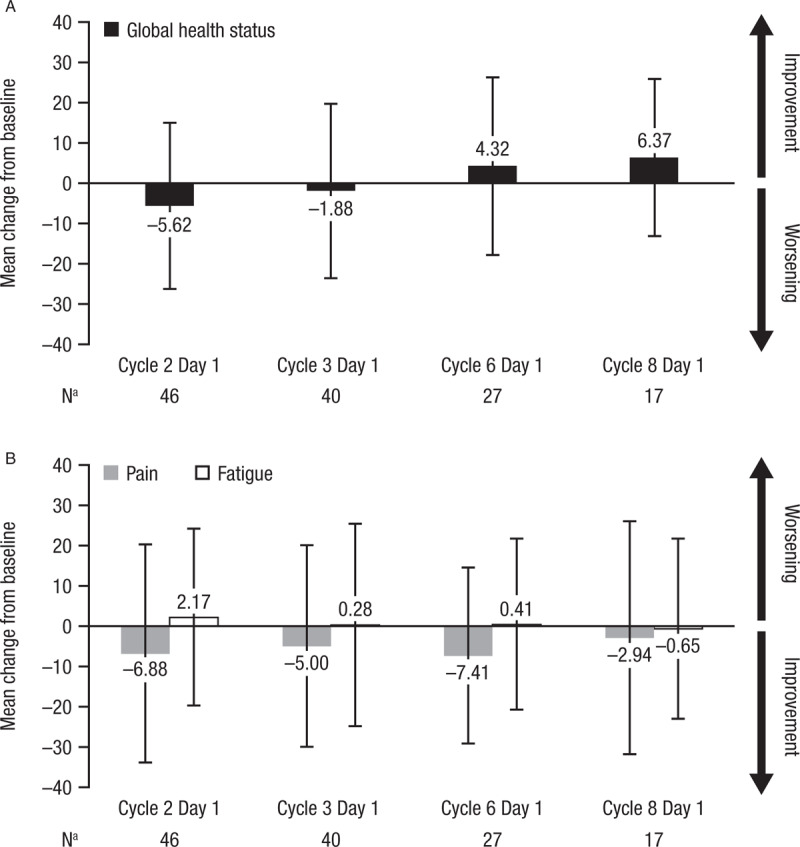
Mean change from baseline (± standard deviation) for global health status (A), and pain and fatigue scores (B) of the European Organisation for Research and Treatment of Cancer (EORTC) Quality of Life (QLQ)-C30 Questionnaire. ^a^The number of patients shown are those who completed the assessment at both baseline and each respective time point.

Mean and median changes from baseline for the European Quality of Life Five Dimensions Questionnaire (EQ-5D-5L) utility score were close to 0 throughout daratumumab treatment (Table 6), and minimal changes from baseline were observed for the EQ-5D-5L visual analog scale (Table 6). The European Organisation for Research and Treatment of Cancer (EORTC) Quality of Life Questionnaire (QLQ)-C30 and EORTC Multiple Myeloma Module (QLQ-MY20) assessments demonstrated that patient functional ability, symptoms, and global health status remained relatively constant throughout daratumumab treatment, with an observed median change from baseline of generally 0 in most domains (Supplemental Digital Content [SDC], Tables 1–3). Mean patient-reported global health status (Fig. 3A) and pain and fatigue symptom scores (Fig. 3B) changed minimally from baseline based on the EORTC QLQ-C30 assessment. Similar patient-reported outcome (PRO) results were seen in patients achieving PR or better.

**Table 6:**
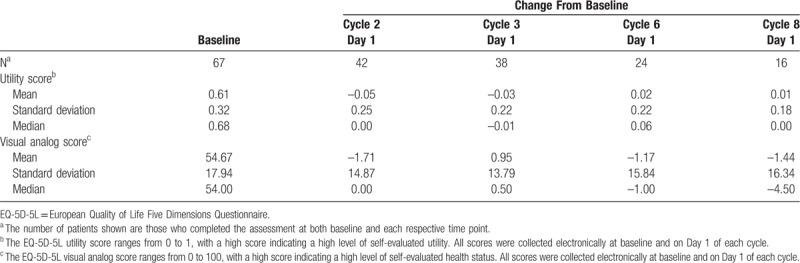
Summary of EQ-5D-5L: Change From Baseline by Visit.

Patients with and without PRO assessments at baseline had comparable baseline characteristics. Responses achieved were also comparable between the 2 groups; however, more patients without PRO assessments had responses that were not evaluable.

## Discussion

MM is a highly heterogeneous disease; clonal heterogeneity increases as the disease progresses, which may lead to varied patient responses to treatment.^24^ The results of this EAP study among Spanish patients with heavily treated (≥3 prior lines of therapy) RRMM confirm the tolerable safety profile of daratumumab monotherapy. The open-label, phase 1/2 GEN501 and phase 2 SIRIUS studies were the first to examine the efficacy and safety profile of daratumumab monotherapy in heavily treated patients with RRMM.^11,12^ The occurrence of grade 3/4 TEAEs (74.0%) in this investigation was similar to that reported in the GEN501 and SIRIUS studies, with lymphopenia (29%), thrombocytopenia (27%), neutropenia (22%), and anemia (15%) being among the most common. SAEs occurred in 47.9% of patients, which is a higher incidence than reported in the SIRIUS study (30%) and may reflect the more heterogeneous patient population in this EAP. The incidence of SAEs reported for US patients participating in this study was 35%^25^; however, patients in this Spanish cohort were exposed to daratumumab for a longer period of time and underwent more daratumumab infusions. IRRs were reported in 33 (45.2%) patients and predominantly occurred during the first infusion, similar to daratumumab clinical trials and the US cohort of this EAP.^25,26^ In addition, the median durations of daratumumab infusions were nearly identical to those observed previously with daratumumab monotherapy.^26^

Although efficacy was not a primary endpoint, an investigator-assessed objective response rate (ORR) of 24.7% (13.7% ≥ VGPR) was observed in this study, similar to the 29.2% overall response rate (12.3% ≥ VGPR) reported in the phase 2 SIRIUS study^12^ and consistent with the 23% ORR (5.5% ≥ VGPR) reported for the US cohort of this EAP study.^25^ The median PFS (3.98 months) was also comparable to that observed in SIRIUS (median PFS: 3.7 months) and the combined analysis of SIRIUS and GEN501 (median PFS: 4.0 months).^12,21^ RRMM patients were heavily treated with ≥3 prior lines of therapy, including PIs and IMiDs, and had evidence of end-organ damage; but despite this, the antitumoral efficacy of daratumumab was remarkably confirmed. Although all patients in the Spanish EAP cohort were heavily treated, no patient had prior exposure to pomalidomide, and no data on refractoriness to prior treatments were collected. Additionally, the short median duration of follow-up, low median number of treatment cycles, and investigator-based assessment of disease response and progression limit the utility of the efficacy data from this study. Upon study closure, there were 14 (19.2%) patients still responding that transitioned to commercially available daratumumab and continued daratumumab treatment provided locally, and there are still 6 patients receiving daratumumab monotherapy as of July 2019.

Since publication of the phase 1/2 GEN501 and phase 2 SIRIUS studies, daratumumab has been shown to be effective and safe in combination with standard-of-care regimens in patients with MM who have received ≥1 prior line of therapy.^13–16^ The phase 3 CASTOR study demonstrated that daratumumab plus bortezomib and dexamethasone reduced the risk of disease progression or death by 61% when compared with bortezomib and dexamethasone alone and drove MM patients to achieve an overall response rate of 83%.^13^ The addition of daratumumab to lenalidomide and dexamethasone in the phase 3 POLLUX trial resulted in a 63% reduction in the risk of disease progression or death and an overall response rate of 93%.^14^ In both studies, responses to daratumumab continued to deepen and were associated with significantly higher minimal residual disease–negativity rates with longer follow-up.^15,16^ Furthermore, these daratumumab-based regimens were well tolerated with similar safety profiles to daratumumab monotherapy.^11–14^ In addition, the phase 3 ALCYONE and MAIA trials recently showed that daratumumab in combination with bortezomib, melphalan, and prednisone or lenalidomide and dexamethasone lowers the risk of disease progression or death by 50% or 44%, respectively, for patients with newly diagnosed MM who are ineligible for stem cell transplantation.^17,18^ More recently, in Part 1 of the phase 3 CASSIOPIEA study, the addition of daratumumab to bortezomib, thalidomide, and dexamethasone during pre-transplant induction and post-transplant consolidation led to increased sCR and minimal residual disease–negativity rates and a 53% reduction in the risk of disease progression or death in transplant-eligible patients with newly diagnosed MM.^19^ Other ongoing phase 3 studies are evaluating daratumumab in combination with bortezomib, lenalidomide, and dexamethasone in newly diagnosed MM patients who are eligible for stem cell transplantation (PERSEUS; NCT03710603) or for whom transplant is not planned as initial therapy (CEPHEUS; NCT03652064).

The deep and durable responses of daratumumab as a monotherapy and across lines of therapy when combined with standard-of-care regimens has led to its approval in many countries for the treatment of MM.^26,27^ However, not all MM patients have access to commercially available daratumumab or ongoing daratumumab clinical trials. The purpose of this investigation was to provide early access to daratumumab for these RRMM patients while collecting additional safety and PRO data. The data from this Spanish cohort of 73 RRMM patients complement the recently reported results for US patients (N = 348) enrolled in this EAP,^25^ and results are forthcoming for additional patient cohorts.

The favorable safety of daratumumab monotherapy in this study was paralleled by maintenance of patient-reported, health-related quality of life, which was quantified using the EQ-5D-5L, EORTC QLQ-C30, and EORTC QLQ-MY20 questionnaires. The baseline EQ-5D-DL utility and visual analog scores for patients with RRMM in this study were within range of what has been previously reported in the US EAP cohort.^25^ The utility score and visual analog scale score changed minimally from baseline to last assessment, suggesting that mobility, self-care, usual activities, pain/discomfort, anxiety/depression, and overall health status remained relatively constant throughout daratumumab treatment. Similarly, EORTC QLQ-C30 and QLQ-MY20 scores changed minimally with daratumumab treatment. The median change from baseline in patient scores of functional ability, symptoms, and global health status was mostly 0, consistent with US patient EORTC QLQ-C30 scores in this study.^25^ Although no substantial improvements in health-related quality of life were noted, minimal change from baseline in these assessments indicate that quality of life was maintained during a median of 3.3 months of daratumumab therapy.

In conclusion, daratumumab monotherapy demonstrated a safety profile in Spanish patients enrolled in the MMY3010 EAP that was consistent with earlier clinical studies of single-agent daratumumab in heavily treated RRMM. The favorable safety profile of daratumumab monotherapy in this EAP was associated with maintenance of patient-reported, health-related quality of life.

## Materials and methods

### Patients

Patients eligible for study participation were ≥18 years of age with documented MM and evidence of disease progression on or after the most recent prior treatment regimen as defined by International Myeloma Working Group (IMWG) criteria; had an ECOG performance status score of 0 to 2; and received ≥3 prior lines of therapy, including a PI and an IMiD, or were double refractory to a PI and an IMiD.^28,29^

The protocol and amendments for this investigation were approved by its sponsor and affiliated local independent ethics committees and internal review boards. All patients provided oral and written consent in accordance with principles that originated in the Declaration of Helsinki, current International Conference on Harmonization and Good Clinical Practice guidelines, applicable regulatory requirements, and sponsor policy.

### Dosing

Daratumumab (16 mg/kg) was administered intravenously every week for 8 weeks (Cycles 1–2), every 2 weeks for 16 weeks (Cycles 3–6), and every 4 weeks thereafter in 28-day cycles until disease progression, lack of clinical benefit, unacceptable toxicity, or study conclusion. Pre- and post-infusion medications were administered on daratumumab infusion days and on the 2 days following infusion to reduce the occurrence of IRRs.

### Assessments and statistical analyses

Patients were monitored continuously for treatment-emergent SAEs (according to the National Cancer Institute Common Terminology Criteria for Adverse Events, version 4.03), grade ≥3 TEAEs, and TEAEs of special interest until 30 days (±7 days) after the last dose of daratumumab at the end of treatment. Vital signs, ECOG performance status, and clinical laboratory parameters were also evaluated, and periodic physical examinations were performed during daratumumab treatment. The safety parameters evaluated during the study included the incidence, severity, and type of TEAEs as well as the relationship of TEAEs to the study drug and any action taken in response to TEAEs.

PROs were assessed using the EQ-5D-5L, EORTC QLQ-C30, and EORTC QLQ-MY20. PRO assessments were collected electronically at baseline; pre-dose Day 1 of Cycles 1, 2, 3, 6, and every other cycle thereafter; and at the end-of-treatment visit. The mean and median changes from baseline for all PRO assessment scores were determined for each patient who completed the assessments at baseline and each respective time point. See SDC, Materials and Methods for additional information.

The analysis population included all patients who received ≥1 dose of daratumumab. SAS software version 9.4 was used for analyzing data. Unless otherwise specified, continuous endpoints were summarized using descriptive statistics, and categorical endpoints were summarized using frequencies and percentages. Exposure to and reasons for discontinuation from study treatment were tabulated. Investigator-assessed disease responses are reported, which were based on IMWG criteria and used to determine whether continued treatment with daratumumab was warranted in accordance with local standard of care as clinically indicated.^30^ The Kaplan-Meier method was used for analysis of PFS, defined as the interval between the first dose of study treatment and either disease progression, as defined by IMWG response criteria, or death, whichever occurred first.

## Acknowledgments

The authors thank the patients who participated in this study, the staff members at the study sites, staff members who were involved in data collection and analyses, and the data and safety monitoring committee. This study was funded by Janssen Research & Development, LLC. Editorial and medical writing support was provided by J. Matthew Kuczmarski, PhD, of MedErgy, and were funded by Janssen Global Services, LLC.

## Sources of Funding

This study was funded by Janssen Research & Development, LLC. Editorial and medical writing support were provided by J. Matthew Kuczmarski, PhD, of MedErgy, and were funded by Janssen Global Services, LLC. The data sharing policy of Janssen Pharmaceutical Companies of Johnson & Johnson is available at https://www.janssen.com/clinical-trials/transparency. As noted on this site, requests for access to the study data can be submitted through the Yale Open Data Access (YODA) Project site at http://yoda.yale.edu.

## Disclosures

AA has served on advisory boards for Janssen, Celgene, Amgen, and Takeda and received research support from Janssen, Celgene, and Amgen. JDLR has received research support from Celgene and Janssen; consulted for AbbVie, Amgen, Celgene, Janssen, and Takeda; and served on advisory boards for Amgen, Celgene, and Janssen. ASB has received travel support from Janssen and served on advisory boards for Janssen, Celgene, and Amgen. CER has no conflicts of interest to disclose. AS has served on advisory boards for Janssen and Celgene. MJB has no conflicts of interest to disclose. JBL has no conflicts of interest to disclose. PR-O has received honoraria derived from lectures and advisory boards from Celgene, Janssen, Bristol-Myers Squibb, and Takeda. AI has no conflicts of interest to disclose. LP has received honoraria from Celgene, Janssen, and Amgen and served on advisory boards for Celgene and Janssen. MJP has received research support from Celgene and served on advisory boards for Amgen, Gilead, Janssen, and Incyte. RR-T has served on advisory boards for Janssen, Celgene, and Amgen. LFCM has received honoraria for lectures from Celgene, Janssen, Roche, Novartis, Bristol-Myers Squibb, Amgen, Takeda, Pfizer, Incyte, and AbbVie and honoraria for participation in advisory boards from Celgene, Janssen, Roche, Novartis, Bristol-Myers Squibb, Amgen, Takeda, Pfizer, Incyte, and AbbVie. MSG has received honoraria from Celgene, Janssen, Takeda, and Amgen. CC was a contracted Janssen employee. AP, HP, HH, IM, and MG are Janssen employees. M-VM has received honoraria for advisory boards and lectures from Janssen, Celgene, Amgen, Takeda, AbbVie, Roche, GlaxoSmithKline, EDO, and PharmaMar.

## Supplementary Material

Supplemental Digital Content
