# Supplementary material for: Results of an Early Access Treatment Protocol of Daratumumab Monotherapy in Spanish Patients With Relapsed or Refractory Multiple Myeloma
Source: Hemasphere. 2020 Jun 3;4(3):e380. doi: 10.1097/HS9.0000000000000380 (PMC7306316; doi:10.1097/HS9.0000000000000380)
Supplement: Supplemental Digital Content [file hs9-4-e380-s001.docx]

**Supplemental Digital Content (SDC) for Alegre et al. Results of an early access treatment protocol of daratumumab monotherapy in Spanish patients with relapsed or refractory multiple myeloma.**

**SDC, MATERIALS AND METHODS**

**European Quality of Life Five Dimensions Questionnaire (EQ-5D-5L)**

The EQ-5D-5L is a 5-item questionnaire that serves as a generic measure of health status and generates utility scores for use in cost-effective analyses. EQ-5D-5L assesses 5 dimensions of health, including mobility, self-care, usual activities, pain/discomfort, and anxiety/depression in addition to a visual analog scale rating overall health.^1^ The 5 dimensions of health are rated by patients on a scale with graded severity that can be used to compute a single utility score. The utility score provides an index of the general health status of each patient, with a maximum score of 1 representing a high level of utility. The visual analog scale rating is selected by patients using an illustration of a health status scale bar ranging from 0 to 100, with 0 being equivocal to the worst health imaginable and 100 being equivocal to the best health imaginable.

**European Organisation for Research and Treatment of Cancer (EORTC) Quality of Life Questionnaire (QLQ)-C30 and Multiple Myeloma Module (QLQ-MY20)**

The EORTC QLQ-C30 is a widely used, clinically meaningful questionnaire that includes 30 questions addressing patient functional ability, symptoms, and overall health status.^2-3^ The EORTC Multiple Myeloma Module (QLQ-MY20) was also administered in conjunction with the EORTC QLQ-C30, and contains an additional 20 questions that are more relevant to myeloma patients.^4^ EORTC QLQ-C30 and QLQ-MY20 scores are summated using the Likert method and presented on a 0 to 100 scale. For global health status and functioning scales, a decrease means a reduction in functioning or utility, whereas a decrease in a symptoms score indicates an improvement in symptoms.

**References**

1. Herdman M, Gudex C, Lloyd A, et al. Development and preliminary testing of the new five-level version of EQ-5D (EQ-5D-5L). *Qual Life Res* 2011; 20(10): 1727-1736.

2. Wisloff F, Hjorth M. Health-related quality of life assessed before and during chemotherapy predicts for survival in multiple myeloma. Nordic Myeloma Study Group. *Br J Haematol* 1997; 97: 29-37.

3. Wisloff F, Eika S, Hippe E, et al. Measurement of health-related quality of life in multiple myeloma. Nordic Myeloma Study Group. *Br J Haematol* 1996; 92(3): 604-613.

4. Cocks K, Cohen D, Wisloff F, et al. An international field study of the reliability and validity of a disease-specific questionnaire module (the QLQ-MY20) in assessing the quality of life of patients with multiple myeloma. *Eur J Cancer* 2007; 43(11): 1670-1678.

| **SDC, Table 1. Summary of EORTC QLQ-C30: Global health status, functioning, and financial difficulties.** | | | | | | | |
| --- | --- | --- | --- | --- | --- | --- | --- |
|  |  | **Change from baseline** | | | | | |
|  | **Baseline** | **Cycle 2 Day 1** | **Cycle 3 Day 1** | **Cycle 6 Day 1** | | **Cycle 8 Day 1** | |
| N^a^ | 68 | 46 | 40 | | 27 | | 17 |
| Global health status |  |  |  | |  | |  |
| Mean | 54.04 | –5.62 | –1.88 | | 4.32 | | 6.37 |
| Standard deviation | 20.34 | 20.64 | 21.64 | | 22.09 | | 19.44 |
| Median | 50.00 | 0.00 | 0.00 | | 0.00 | | 8.33 |
| Functioning |  |  |  | |  | |  |
| Cognitive |  |  |  | |  | |  |
| Mean | 80.15 | 1.09 | 0.42 | | 0.62 | | 1.96 |
| Standard deviation | 24.81 | 17.71 | 13.86 | | 23.79 | | 15.46 |
| Median | 83.33 | 0.00 | 0.00 | | 0.00 | | 0.00 |
| Emotional |  |  |  | |  | |  |
| Mean | 68.01 | 6.88 | 2.08 | | 0.31 | | 3.92 |
| Standard deviation | 24.42 | 15.84 | 24.15 | | 20.61 | | 15.90 |
| Median | 75.00 | 4.17 | 0.00 | | 0.00 | | 8.33 |
| Physical |  |  |  | |  | |  |
| Mean | 64.71 | –4.93 | 0.17 | | –1.23 | | 2.35 |
| Standard deviation | 24.25 | 20.77 | 17.96 | | 16.33 | | 15.45 |
| Median | 66.67 | 0.00 | 0.00 | | 0.00 | | 6.67 |
| Role |  |  |  | |  | |  |
| Mean | 57.35 | –6.16 | –1.67 | | –2.47 | | 1.96 |
| Standard deviation | 33.63 | 29.68 | 30.85 | | 22.98 | | 29.98 |
| Median | 66.67 | 0.00 | 0.00 | | 0.00 | | 0.00 |
| Social |  |  |  | |  | |  |
| Mean | 62.25 | 2.90 | 1.25 | | 6.79 | | 11.76 |
| Standard deviation | 30.42 | 32.45 | 27.83 | | 25.43 | | 22.64 |
| Median | 66.67 | 0.00 | 0.00 | | 0.00 | | 0.00 |
| Financial difficulties |  |  |  | |  | |  |
| Mean | 20.59 | –9.42 | –2.50 | | –3.70 | | –5.88 |
| Standard deviation | 29.38 | 27.81 | 19.08 | | 16.88 | | 21.20 |
| Median | 0.00 | 0.00 | 0.00 | | 0.00 | | 0.00 |

EORTC QLC-C30, European Organisation for Research and Treatment of Cancer Quality of Life Questionnaire-C30.

^a^The number of patients shown are those who completed the assessment at both baseline and each respective time point.

| **SDC, Table 2. Summary of EORTC-QLQ-C30: patient-reported symptoms.** | | | | | | | | | | |
| --- | --- | --- | --- | --- | --- | --- | --- | --- | --- | --- |
|  |  |  | | **Change from baseline** | | | | | | |
|  | **Baseline** | | **Cycle 2 Day 1** | | | **Cycle 3 Day 1** | | **Cycle 6 Day 1** | | **Cycle 8 Day 1** |
| N^a^ | 68 | | 46 | | | 40 | | 27 | | 17 |
| Appetite loss |  | |  | |  | |  | |  | |
| Mean | 21.57 | | 5.07 | | | 4.17 | | 0.00 | | –7.84 |
| Standard deviation | 30.35 | | 35.81 | | | 21.60 | | 29.24 | | 32.34 |
| Median | 0.00 | | 0.00 | | | 0.00 | | 0.00 | | 0.00 |
| Constipation |  | |  | | |  | |  | |  |
| Mean | 17.16 | | 0.72 | | | 1.67 | | 6.17 | | –5.88 |
| Standard deviation | 30.20 | | 28.54 | | | 18.41 | | 22.72 | | 13.10 |
| Median | 0.00 | | 0.00 | | | 0.00 | | 0.00 | | 0.00 |
| Diarrhea |  | |  | | |  | |  | |  |
| Mean | 18.14 | | –2.17 | | | –5.83 | | 0.00 | | –5.88 |
| Standard deviation | 31.25 | | 21.55 | | | 26.03 | | 16.01 | | 13.10 |
| Median | 0.00 | | 0.00 | | | 0.00 | | 0.00 | | 0.00 |
| Dyspnea |  | |  | | |  | |  | |  |
| Mean | 18.14 | | –4.35 | | | 0.00 | | 8.64 | | –7.84 |
| Standard deviation | 25.39 | | 29.49 | | | 22.65 | | 27.10 | | 22.14 |
| Median | 0.00 | | 0.00 | | | 0.00 | | 0.00 | | 0.00 |
| Fatigue |  | |  | | |  | |  | |  |
| Mean | 44.61 | | 2.17 | | | 0.28 | | 0.41 | | –0.65 |
| Standard deviation | 27.52 | | 21.93 | | | 25.10 | | 21.24 | | 22.39 |
| Median | 44.44 | | 0.00 | | | 0.00 | | 0.00 | | 0.00 |
| Nausea and vomiting |  | |  | | |  | |  | |  |
| Mean | 5.39 | | 1.09 | | | 1.67 | | 3.09 | | –2.94 |
| Standard deviation | 14.24 | | 13.79 | | | 12.40 | | 15.36 | | 14.71 |
| Median | 0.00 | | 0.00 | | | 0.00 | | 0.00 | | 0.00 |
| Pain |  | |  | | |  | |  | |  |
| Mean | 48.77 | | –6.88 | | | –5.00 | | –7.41 | | –2.94 |
| Standard deviation | 30.92 | | 27.12 | | | 25.09 | | 21.85 | | 29.01 |
| Median | 50.00 | | 0.00 | | | 0.00 | | 0.00 | | 0.00 |
| Sleep disturbance |  | |  | | |  | |  | |  |
| Mean | 30.39 | | 1.45 | | | 0.00 | | 0.00 | | -9.80 |
| Standard deviation | 32.95 | | 32.93 | | | 32.03 | | 26.15 | | 22.87 |
| Median | 33.33 | | 0.00 | | | 0.00 | | 0.00 | | 0.00 |

EORTC QLC-C30, European Organisation for Research and Treatment of Cancer Quality of Life Questionnaire-C30.

^a^The number of patients shown are those who completed the assessment at both baseline and each respective time point.

| **SDC, Table 3. Summary of EORTC QLQ-MY20.** | | | | | | | | | | | |  |
| --- | --- | --- | --- | --- | --- | --- | --- | --- | --- | --- | --- | --- |
|  |  |  | | **Change from baseline** | | | | | | | |  |
|  | **Baseline** | | **Cycle 2 Day 1** | | | **Cycle 3 Day 1** | | **Cycle 6 Day 1** | | **Cycle 8 Day 1** | | |
| N^a^ | 69 | | 45 | | | 40 | | 26 | | 17 | | |
| Body image |  | |  | |  | |  | |  | |  |  |
| Mean | 71.01 | | 2.22 | | | 0.83 | | 5.13 | | 7.84 | | |
| Standard deviation | 35.20 | | 26.02 | | | 27.72 | | 26.15 | | 18.74 | | |
| Median | 100.00 | | 0.00 | | | 0.00 | | 0.00 | | 0.00 | | |
| Disease symptoms |  | |  | | |  | |  | |  | | |
| Mean | 33.98 | | –2.84 | | | –5.00 | | –1.92 | | –5.56 | | |
| Standard deviation | 23.75 | | 19.38 | | | 23.66 | | 19.43 | | 18.11 | | |
| Median | 33.33 | | 0.00 | | | 0.00 | | –2.78 | | 0.00 | | |
| Future perspective |  | |  | | |  | |  | |  | | |
| Mean | 57.97 | | 5.68 | | | 2.22 | | 7.69 | | 5.23 | | |
| Standard deviation | 26.40 | | 22.30 | | | 26.05 | | 22.39 | | 18.05 | | |
| Median | 66.67 | | 0.00 | | | 0.00 | | 11.11 | | 11.11 | | |
| Side effects of treatment |  | |  | | |  | |  | |  | | |
| Mean | 24.07 | | –0.22 | | | –2.37 | | 0.63 | | –1.02 | | |
| Standard deviation | 16.91 | | 12.36 | | | 13.85 | | 12.19 | | 11.74 | | |
| Median | 22.22 | | 0.00 | | | 0.00 | | 0.00 | | –3.70 | | |

EORTC QLC-MY20, European Organisation for Research and Treatment of Cancer Quality of Life Questionnaire-C30 and Multiple Myeloma Module.

^a^The number of patients shown are those who completed the assessment at both baseline and each respective time point.
